# Inspiratory effort increases blood volume in the thoracic cavity and decreases end-expiratory lung impedance: a preliminary prospective study

**DOI:** 10.1007/s00421-025-05767-5

**Published:** 2025-04-03

**Authors:** Kazuhiro Takahashi, Ayaka Koyama, Daisuke Irimada, Akihiro Kanaya, Daisuke Konno, Yu Kaiho, Yusuke Takei, Kazutomo Saito, Yutaka Ejima, Masanori Yamauchi

**Affiliations:** Department of Anesthesiology and Perioperative Medicine, Tohoku University Graduate School of Medicine, Sendai, Japan

**Keywords:** Electrical impedance tomography (EIT), End-expiratory lung impedance (EELI), Airway resistance, Thoracic cavity, Blood volume, Inspiratory effort

## Abstract

**Purpose:**

Passive leg raising (PLR) increases intrathoracic blood volume by redistributing blood from the lower to the upper body area. While inspiratory effort is hypothesized to have a similar effect due to pressure differences between the intrathoracic and extrathoracic cavities, direct evidence is scarce. Therefore, this study evaluated whether excessive inspiratory effort increases intrathoracic blood volume using end-expiratory lung impedance (EELI).

**Methods:**

Volunteers, fitted with electrical impedance tomography (EIT) belts, underwent a spontaneous breathing procedure in the supine position (control step). They breathed through a specialized face mask with separated inspiration and expiration routes (one-way valves) and their EELI was continuously recorded. First, PLR was performed. Subsequently, resistors (3-mm and 2-mm) were sequentially added to the mask’s inspiration route, requiring volunteers to increase inspiratory effort. A reference EELI was established during spontaneous breathing, and changes in EELI (ΔEELI) were calculated for each step (control, PLR, 3-mm, and 2-mm). ΔEELI values were compared using the Friedman test and Wilcoxon signed-rank test with Holm’s *P* value adjustment.

**Results:**

Across 11 participants, the mean ΔEELI decreased by 13, 18, and 19 units for PLR, 3-mm, and 2-mm resistors, respectively. The Friedman test and Wilcoxon signed-rank test revealed significant differences between the control and each aforementioned intervention.

**Conclusion:**

PLR and increased inspiratory effort augment thoracic blood volume, thereby reducing EELI.

**Registration:**

UMIN000054238. April/23/2024.

**Supplementary Information:**

The online version contains supplementary material available at 10.1007/s00421-025-05767-5.

## Background

Electrical impedance tomography (EIT) is a noninvasive technique used to assess the thoracic cavity. Because blood conducts electricity more effectively than air, the impedance is higher at the end of inspiration than at the end of expiration. EIT signals provide information on tidal volume, functional residual capacity (FRC), and ventilation uniformity.

Among the various EIT parameters, end-expiratory lung impedance (EELI) has attracted significant attention in recent years. Changes in the EELI are highly correlated with air volume (Bikker et al. 2009; Spooner et al. 2014) and are therefore considered an alternative estimate of end-expiratory lung volume (EELV) (Hinz et al. 2003).

Furthermore, the amount of blood and electrolyte concentration in a patient’s body alters thoracic impedance (Adler et al. 1997). Under constant ventilation, transfusion decreases EELI, whereas blood withdrawal increases it (Bodenstein et al. 2012; Becher et al. 2019).

We hypothesized that changes in blood distribution in the body (blood transfer into the thoracic cavity from other parts of the body) may affect the EELI. For example, passive leg raising (PLR) is thought to increase blood transfer into the thoracic cavity and the amount of blood in it (He and Liu 2016).

Similarly, inspiratory effort may increase blood transfer because it decreases intrathoracic pressure (ITP) (Pinsky 2018). The pressure difference between the intrathoracic and extrathoracic cavities is the driving force for blood transfer into the thoracic cavity.

Although the mechanism of blood transfer by inspiratory effort is intuitively clear, evidence has only been indirectly indicated by an increase in arterial blood pressure or cardiac output (flow rate) (Convertino et al. 2011; Skytioti and Søvik 2018; Guérin et al. 2015). In other words, no study has examined whether inspiratory efforts increase blood volume in the thoracic cavity (volume) by facilitating blood transfer into the thoracic cavity and retaining that blood. Determining whether inspiratory effort increases blood content in the thoracic cavity is crucial, as this is directly related to theories often used in clinical practice, such as negative pressure pulmonary edema during upper airway obstruction and pulmonary edema associated with patient self-inflicted lung injury (P-SILI).

This study aimed to detect intrathoracic blood volume changes caused by PLR and inspiratory effort using EELI. We hypothesized that EELI would decrease after PLR and strong inspiratory effort and then smoothly return to control once these actions were discontinued.

## Methods

This prospective volunteer study was conducted at the Tohoku University Hospital following approval from the Ethics Committee of the Tohoku University Graduate School of Medicine. (UMIN number: UMIN000054238). Written informed consent was obtained from all participants before enrollment.

### Participants

The participants were healthy adults with an American Society of Anesthesiologists physical status (ASA-PS) of 1 or 2, and written consent to participate in the study was obtained. Participants with a history of respiratory, cardiovascular, or neuromuscular disease were excluded.

### Measurement

After registration, an appropriately sized EIT belt (Enlight 2100; Medtronic, Dublin, Ireland) was wrapped around the participant’s chest between the fourth and sixth intercostal spaces and connected to the EIT device. The EIT and pressure/flow rate data were acquired at a sampling frequency of 50 Hz for each measurement.

**Fig. 1:**
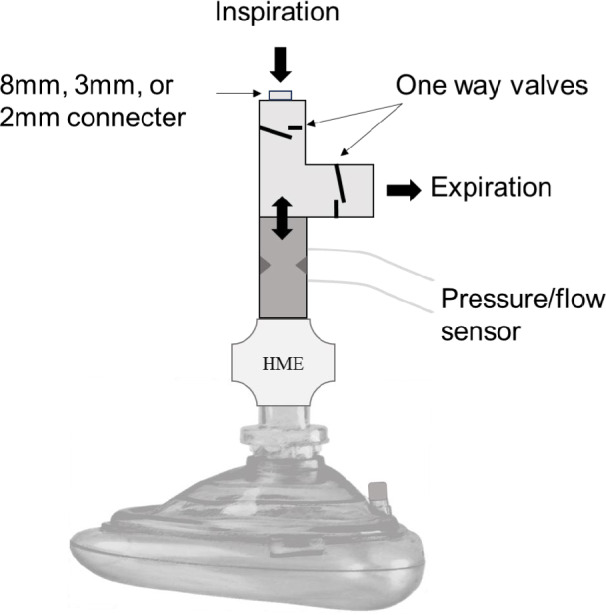
Specialized face mask used in the study. A face mask with two built-in one-way valves, a heat and moisture exchanger (HME), and a pressure/flow sensor. The tip of the face mask was branched into two halves, each connected to a one-way valve designed to separate the inhalation and exhalation routes of the participants. The tip on the inhalation side has an 8-mm (control), 3-mm, or 2-mm connector, and the tip on the exhalation side does not

During the measurements, a specialized face mask with an integrated pressure/flow sensor (Enlight 2100) was fitted onto the face of the participant without air leakage. The end of the face mask was branched into two halves, each connected to a one-way valve designed to separate the inhalation and exhalation routes (Fig. 1). An 8-mm (for the control step), 3-mm, or 2-mm internal diameter (ID) connector was connected to the inhalation side, whereas no resistor was connected to the exhalation side (11.5 mm ID). Because the 3- or 2-mm ID connector functions as a higher resistor than the 8-mm ID connector, a strong inspiratory effort is required to sufficiently inhale through the specialized facemask with the 3- or 2-mm ID connectors.

The participants were instructed to breathe at a constant rhythm (3 s and 6 s for inhalation and exhalation, respectively) using a metronome. For inhalation, the participants were instructed to inhale as much as they wanted without being aware of the tidal volume. For exhalation, the participants were directed to exhale within 5 s and rest for the last 1 s (exhalation within 5 s was easy because no resistance was connected to the exhalation side). Therefore, before the next inspiration, the lung air volume is presumed to return to the FRC, and the respiratory muscles relax.

**Fig. 2:**
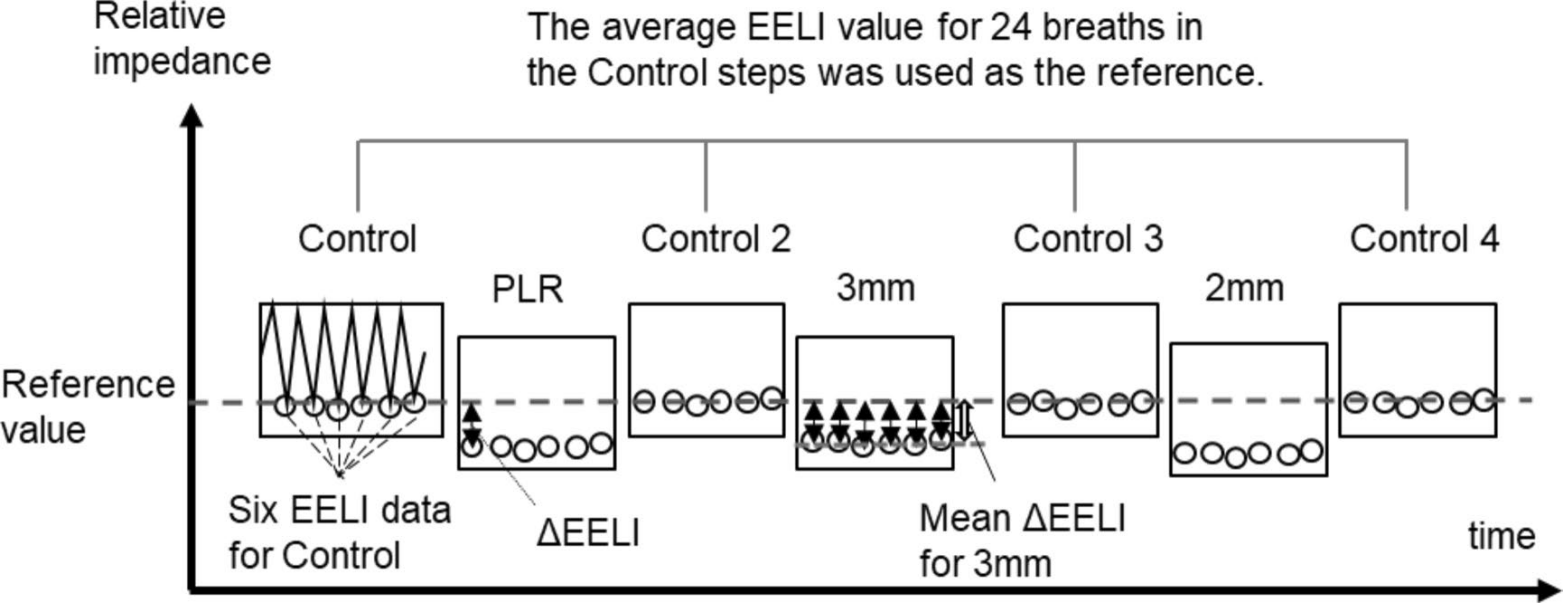
Schematic of measurements. Schematic representation of the measurements performed for each participant. The vertical axis shows the relative impedance values, and the horizontal axis shows the time. The measurements consisted of control and intervention steps, with six breaths per step. All steps were performed consecutively. The average EELI value for 24 breaths during the control steps was used as the reference. The difference between this reference value and the EELI for each breath was ΔEELI, and the average of the six ΔEELIs for each step was calculated. PLR, passive leg raising; 3 mm, inspiratory effort intervention by ID 3-mm resistor; 2 mm, inspiratory effort intervention by ID 2-mm resistor

Measurements were performed in the supine position. After spontaneous breathing was stabilized, EIT measurements with the aforementioned face mask began using an 8-mm connector (control step). Three types of interventions (PLR, 3-mm resistor, and 2-mm resistor) were conducted. The PLR was initiated in the supine position, and both legs were lifted to 60° (He and Liu 2016). A control step was performed after each intervention (Fig. 2). The participants were informed of this in advance.

Six consecutive breaths were measured at each step, and the relative impedance values of the EIT, airway pressure, and flow rates were recorded.

### Statistical analysis

Owing to the lack of publicly available data at the time of this study, a power analysis could not be performed. Therefore, we recruited 11 participants based on previous studies that have focused on EELI (Hinz et al. 2003; Sobota et al. 2019).

The analysis was performed using EIT Analysis (Medtronic) software dedicated to the Enlight 2100. The smallest relative impedance value for each respiratory cycle was extracted as EELI. The EELI, which is the sum of the entire pixel impedance at the end of expiration, was analyzed offline after the measurement. The mean EELI of all control steps was used as the reference value for each participant, and the difference between the measured value and each participant’s reference value was used for statistical analysis (ΔEELI).

The ΔEELI during the first control step and the three types of interventions were compared using the Friedman test. Multiple comparisons were performed using the Wilcoxon signed-rank test (with the Holm *P* value adjustment method).

The mean airway pressure was calculated for each step. Subsequently, the Friedman test and the Wilcoxon signed-rank test with the *P* value adjustment method of Holm were conducted.

Statistical significance was set at *P* < 0.05.

## Results

All relevant data are presented in this paper, as well as in the Supporting Information files. The profiles of the volunteers are presented in Table 1. No applications for participation were received from women, possibly because the EIT band had to be attached to the frontal chest. The body mass index of the participants ranged from 17.7 to 26.9. No unforeseen events occurred during the measurement period, and no complaints of poor health were reported. The mask fit was adequate for all participants, and all participants completed the data acquisition. This was a preliminary study, and the only data obtained were EIT data, pressure, and flow rate data.

**Table 1:** Profiles of the participants

Age (y), median [IQR]	32 [29, 38]
Sex (male/female)	11/0
Height (cm), median [IQR]	170 [167, 173]
Weight (kg), median [IQR]	68 [65, 73]
BMI, median [IQR]	23.2 [21.7, 26.8]
Belt size,* n* (%)	
XS	2 (18.2%)
S	5 (45.5%)
M	4 (36.3%)

*BMI* body mass index; *IQR* interquartile range

**Fig. 3:**
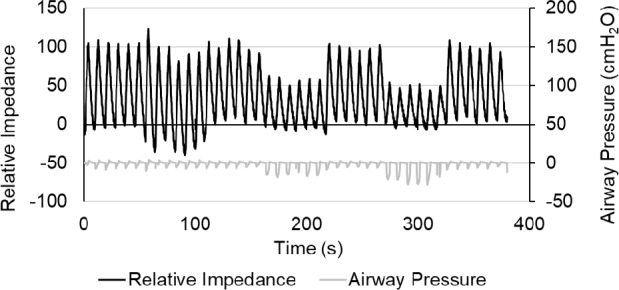
Representative traces of lung impedance and airway pressure. Representative traces of lung impedance (upper black graph, left vertical axis) and airway pressure (lower gray graph, right vertical axis) during the seven steps. The horizontal axis represents time (s). From left to right, control and intervention steps (control, PLR, control, 3-mm resistor, control, 2-mm resistor, control) were consecutively performed

Figure 3 presents representative traces of impedance (upper graph, left vertical axis) and airway pressure (lower graph, right vertical axis) during the measurements (Participant No. 10). PLR and inspiratory effort interventions promptly altered the EELI. Owing to the respiratory variability of spontaneous breathing, the impedance values at each step were not perfectly consistent. As expected, the airway pressure decreased during inhalation through the 3- and 2-mm connectors. Furthermore, the maximal inspiratory flow rate decreased when inhaled through these connectors. Online Resource 1 shows the mean EELI, mean airway pressure, and maximal inspiratory flow rate for each participant at every step.

**Fig. 4:**
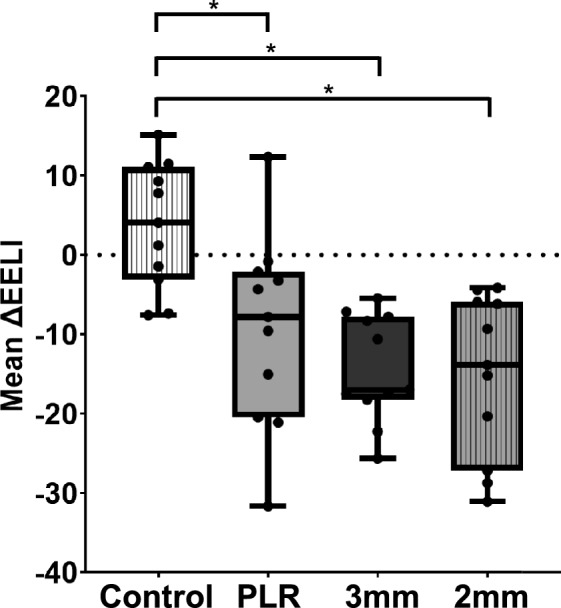
Mean ΔEELI during the first control step and the three types of interventions. From left to right, the four box whiskers indicate the first control step (white monochrome), PLR (gray monochrome), 3 mm (black monochrome), and 2 mm (narrow vertical stripes on a gray background). Each box whisker represents the interquartile range (IQR), which contains the middle 50% of the data. The line inside the box indicates the median and the whiskers show the rest of the distribution, excluding outliers (points beyond whiskers). Black circles indicate data from individual participants. * denotes a significant difference (*P* < 0.05)

Figure 4 shows the mean ΔEELI during the first control step and the three types of interventions. Compared with the control step, PLR and inspiratory effort reduced ΔEELI. The mean ΔEELI decreased by 13, 18, and 19 units for the PLR, 3 mm resistor, and 2-mm resistor, respectively, compared with that in the control step. A significant difference was detected using the Friedman test (*P* = 0.00391). Multiple comparisons revealed significant differences between the control and PLR (*P* = 0.0097), control and 3 mm (*P* = 0.0059), and control and 2 mm (*P* = 0.0391) groups. Online Resource 2 provides an intuitive figure that facilitates the EELI changes before and after each intervention.

**Fig. 5:**
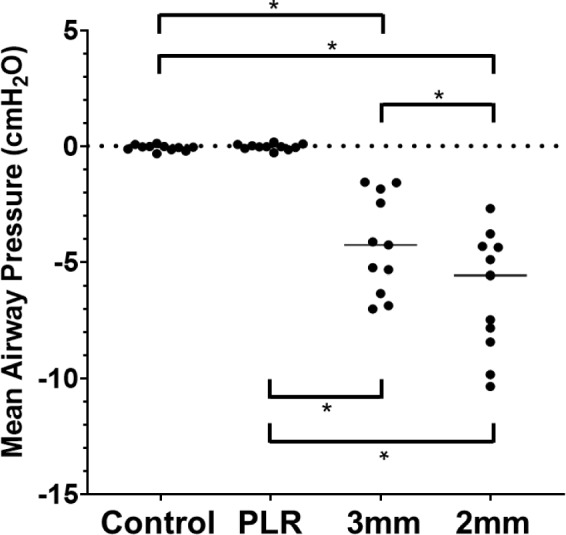
Mean airway pressure during the first control step and the three types of interventions. The figure shows the mean airway pressure during the trials. Each point represents the mean airway pressure at each step for the 11 participants (from left to right: control, PLR, 3 mm, and 2 mm). * denotes a significant difference (*P* < 0.05)

Figure 5 shows the mean airway pressure during the first control step and intervention. During the control step, the mean airway pressure was approximately zero. Similarly, the mean airway pressure was clustered around zero during PLR, which is reasonable because PLR is thought to have little effect on spontaneous breathing. In contrast, during inhalation through the 3- or 2-mm connectors, the mean airway pressure decreased. Significant differences between the four steps were detected using the Friedman test (*P* = 0.0000024). Multiple comparisons revealed significant differences between the control and 2-mm resistors (*P* = 0.0059), control and 3-mm resistors (*P* = 0.0059), PLR and 3-mm resistors (*P* = 0.0059), PLR and 2-mm resistors (*P* = 0.0059), and 3-mm and 2-mm resistors (*P* = 0.0293). However, no significant differences were observed between the control and PLR groups (*P* = 1.0000).

## Discussion

### PLR and inspiratory effort increase blood volume in the thoracic cavity

This volunteer study was conducted to detect intrathoracic blood volume changes caused by PLR and inspiratory effort using EIT. As expected, PLR and inspiratory effort increased the intrathoracic blood volume and reduced the EELI.

PLR undoubtedly increases blood volume in the thoracic cavity. As blood moves according to gravity, a decrease in blood in both legs indicates an increase in blood in other areas of the body.

As for inspiratory effort, the relationship between spontaneous breathing and venous return has been studied (Pinsky 2018; Eckstein and Hamilton 1958). Studies have shown that strong spontaneous breathing or negative ITP increases venous return and cardiac output (Convertino et al. 2011; Yoshida et al. 2017; Hagiwara-Nagasawa et al. 2021). The pressure difference between the intrathoracic and extrathoracic cavities is the driving force of this blood flow. If the inspiratory effort is strong, this pressure difference is expected to increase. Some studies have forcefully created this pressure difference to increase cardiac output (Hagiwara-Nagasawa et al. 2021; Birch et al. 2015).

However, although the concepts of blood volume within the thoracic cavity and cardiac output are related, they are fundamentally different. No studies have focused on whether excessive inspiratory effort retains blood in the thoracic cavity. To date, an increase in blood volume in the thoracic cavity has only been indirectly indicated by a decrease in intravascular wall pressure (Yoshida et al. 2017; Mauri et al. 2016). To address this lack of evidence, EIT is appropriate owing to its fine temporal resolution. For example, using EIT in line with this concept, a previous animal study showed that venous return restriction using a balloon catheter increased thoracic impedance (Fagerberg et al. 2009).

In this study, EELI decreased quickly after PLR and inspiratory effort and smoothly returned to baseline after these interventions were discontinued (Online Resource 2). This increase in EELI is presumed to be mainly due to changes in pulmonary vascular filling because pulmonary edema is not expected to return to the baseline promptly. In addition, as no transfusions were performed, electrolyte changes likely did not affect the EIT measurements.

In this study, inhalation and exhalation were designed to use different routes by combining two one-way valves. The resistor was then attached to the inhalation side to reduce the ITP through spontaneous breathing (Convertino et al. 2011). The idea of using resistance during inhalation is not unusual, and some studies have used an impedance threshold device originally intended for use during cardiopulmonary resuscitation (James et al. 2015; Sigurdsson et al. 2006). These devices demonstrate that inspiratory effort lowers ITP. If airway resistance is low, air enters the thoracic cavity; if airway resistance is high, blood enters the thoracic cavity. Online Resource 1 indicates inspiratory flow restriction and airway pressure decrease during the 3-mm and 2-mm resistor interventions.

### How does this research apply to clinical practice?

The physiological finding in this study is that inspiratory effort increases the blood volume in the thoracic cavity, which is important in terms of the interaction between respiration and circulation. If sufficient air does not flow into the thoracic cavity despite the person’s inspiratory effort (due to airway resistance), blood flows into the thoracic cavity. This blood flow increases venous return and blood pressure, which is beneficial from a circulatory perspective. However, from the perspective of respiration, prolonged increased blood volume in the thoracic cavity can lead to pulmonary edema.

This study demonstrated that the combination of elevated airway resistance and intense inspiratory effort serves as a mechanism that facilitates blood transfer into the thoracic cavity and retains that blood. Numerous factors contribute to airway resistance in clinical practice. For instance, research has demonstrated that tracheal intubation doubles airway resistance compared to a patient’s natural airway. (Takahashi et al. 2023a). The thinner the ETT, the greater the resistance (Takahashi et al. 2023b). A high gas density also increases airway resistance (Takahashi et al. 2022). Heat and moisture exchangers (HME) also act as resistors, and their pressure drops depend on the flow rate (Arieli et al. 2011). Furthermore, there is variability in its resistance across different HME products, and that resistance increases upon exposure to moisture. (Lucato et al. 2005).

In contrast, strong inspiratory effort is frequently observed in patients with acute respiratory distress syndrome, and lung injury associated with this intense inspiratory effort is recognized as P-SILI. In P-SILI, excessive inspiratory effort is thought to increase the pressure gradient inside and outside the pulmonary vessels, and some of the water leaking from the pulmonary vessels seeps into the alveoli, causing pulmonary edema (Yoshida et al. 2017). Negative-pressure pulmonary edema can be explained by a similar mechanism resulting from strong spontaneous breathing with an obstructed or narrowed airway (Ma et al. 2023).

In addition, this study provides new insights into the relationship between spontaneous breathing trials (SBT) and EELI. Previous studies have suggested that a decrease in ΔEELI in the SBT failure group reflects a reduction in EELV (Wisse et al. 2024; Longhini et al. 2019; Lima et al. 2019; Zhao et al. 2017). We concur with this perspective; however, the increase in intrathoracic blood content caused by inspiratory effort warrants further attention.

Because EIT parameters vary with the interaction between respiration and circulation, it is difficult to make a clinical decision based on EIT alone. However, its usefulness may increase when combined with other parameters. For example, in a fixed ventilator setting, if the cardiac output and blood pressure increase during infusion loading while the EIT impedance decreases overall, we would consider the possibility of pulmonary congestion due to excess infusion. In addition, under a fixed ventilator setting, if there is an overall decrease in EIT impedance along with a decrease in esophageal pressure, there may be an increase in airway resistance due to sputum in the tracheal tube or water adherence to the HME, resulting in increased inspiratory effort and intrathoracic blood volume. Therefore, combining EIT data with other parameters, such as ventilator graphics, esophageal pressure, and blood pressure, may allow for more plausible interpretations.

### Limitations

A notable limitation of this study is that EELV was not directly measured in the participants. This is a common limitation of spontaneous breathing studies that use EIT. Therefore, we devised a method to maintain the FRC as much as possible. The resistance applied to the inspiratory side did not affect expiration when a special mask with separate inspiratory and expiratory routes was used. Furthermore, by allowing adequate time for exhalation and rest at the end of expiration, the participant’s lung air volume was closer to that of the FRC. Consequently, the participant promptly returned to the baseline after the 3-mm and 2-mm resistance steps, suggesting that atelectasis was unlikely to occur.

The direct evaluation of EELV is challenging in practice. Plethysmographs, which are a typical method of measuring FRC, assume that the patient’s oral and alveolar pressure changes are in agreement and are not suitable for inhalation with a resistance-attached mask, as in this study. Although radiography and computed tomography may be useful, they do not provide sufficient time resolution.

In addition, because this study was conducted on conscious, spontaneously breathing volunteers, respiratory variability was observed (van den Bosch et al. 2021). Respiratory variability is influenced by multiple factors, including sleep, various pulmonary diseases, hypoxia, and anxiety disorders, and it is difficult to remove this variability from a conscious person altogether. Therefore, we attempted to minimize this variability as much as possible by making participants aware of their breathing rhythms. Previous studies have reported that the respiratory rate (RR) is primarily modulated by central command and muscle afferent feedback, whereas metabolic inputs modulate the tidal volume (TV). (Nicolò and Sacchetti 2023). From this perspective, respiratory variability was minimized in this study, as the RR was regulated using a metronome, and the intervention was exceedingly brief; therefore, the impact on the metabolic input was considered negligible.

Another limitation was the absence of female participants. However, sex differences are unlikely to have influenced the results of this study.

### Principles for future research

This study served as a preliminary investigation, and only minimal parameters were obtained to demonstrate that inspiratory effort facilitates blood transfer into the thoracic cavity and blood retention. By combining EIT with other methodologies, substantive information can be acquired (Rocha et al. 2024). For instance, cardiac output can be measured invasively by pulmonary artery catheter thermodilution and arterial pressure-based cardiac output (APCO) (McGee et al. 2007) and noninvasively by echocardiography (Zhang et al. 2019). The blood volume in the thoracic cavity can be measured using the indicator dilution method (Schreiber et al. 2001). Esophageal pressure provides useful information as a surrogate for ITP (Yoshida et al. 2017). Similarly, additional methodologies may be required to ensure accurate lung-air-volume measurements. For instance, a technique analogous to the helium dilution method, such as connecting a patient's airway to a bag of known volume, could be employed.

## Conclusions

This study demonstrated that the PLR and inspiratory effort decreased the EELI, indicating an increase in the intrathoracic blood volume. These findings enhance our understanding of cardiopulmonary interactions and may have implications for clinical practice. Despite its limitations, particularly the lack of direct EELV measurements, this study provides valuable insights and a foundation for future research in this important field.

## Supplementary Information

Below is the link to the electronic supplementary material.

Supplementary file1 (PDF 203 KB)

Supplementary file2 (PDF 186 KB)

## Data Availability

All relevant data have been included in the manuscript and its supporting information files.

## Abbreviations

ASA-PS: American society of anesthesiologists-physical status
EELI: End-expiratory lung impedance
EELV: End-expiratory lung volume
EIT: Electrical impedance tomography
ETT: Endotracheal tube
FRC: Functional residual capacity
ID: Internal diameter
ITP: Intrathoracic pressure
PLR: Passive leg raising
P-SILI: Patient self-inflicted lung injury
SBT: Spontaneous breathing trial

## References

[CR1] Adler A, Amyot R, Guardo R, Bates JH, Berthiaume Y (1997) Monitoring changes in lung air and liquid volumes with electrical impedance tomography. J Appl Physiol 83:1762–1767. 10.1007/s10877-023-01038-w 9375349 10.1152/jappl.1997.83.5.1762

[CR2] Arieli R, Daskalovic Y, Ertracht O, Arieli Y, Adir Y, Abramovich A, Halpern P (2011) Flow resistance, work of breathing of humidifiers, and endotracheal tubes in the hyperbaric chamber. Am J Emerg Med 29:725–730. 10.1152/jappl.1997.83.5.1762 20825878 10.1016/j.ajem.2010.02.003

[CR3] Becher T, Wendler A, Eimer C, Weiler N, Frerichs I (2019) Changes in electrical impedance tomography findings of ICU patients during rapid infusion of a fluid bolus: a prospective observational study. Am J Respir Crit Care Med 199:1572–1575. 10.1164/rccm.201812-2252LE 30875244 10.1164/rccm.201812-2252LE

[CR4] Bikker IG, Leonhardt S, Bakker J, Gommers D (2009) Lung volume calculated from electrical impedance tomography in ICU patients at different PEEP levels. Intensive Care Med 35:1362–1367. 10.1007/s00134-009-1512-6 19513694 10.1007/s00134-009-1512-6 PMC2712617

[CR5] Birch M, Kwon Y, Loushin MK, Puertas L, Prielipp R, Belani K, Beebe D (2015) Intrathoracic pressure regulation to treat intraoperative hypotension: a phase II pilot study. Eur J Anaesthesiol 32:376–380. 10.1097/EJA.0000000000000234 25946059 10.1097/EJA.0000000000000234

[CR6] Bodenstein M, Wang H, Boehme S, Vogt A, Kwiecien R, David M, Markstaller K (2012) Influence of crystalloid and colloid fluid infusion and blood withdrawal on pulmonary bioimpedance in an animal model of mechanical ventilation. Physiol Meas 33:1225–1236. 10.1088/0967-3334/33/7/1225 22735353 10.1088/0967-3334/33/7/1225

[CR7] Convertino VA, Ryan KL, Rickards CA, Glorsky SL, Idris AH, Yannopoulos D, Metzger A, Lurie KG (2011) Optimizing the respiratory pump: harnessing inspiratory resistance to treat systemic hypotension. Respir Care 56:846–857. 10.4187/respcare.01018 21333089 10.4187/respcare.01018 PMC6607101

[CR8] Eckstein JW, Hamilton WK (1958) Changes in transmural central venous pressure in man during hyperventilation. J Clin Invest 37:1537–1541. 10.1172/JCI103745 13587662 10.1172/JCI103745 PMC1062835

[CR9] Fagerberg A, Stenqvist O, Åneman A (2009) Monitoring pulmonary perfusion by electrical impedance tomography: an evaluation in a pig model. Acta Anaesthesiol Scand 53:152–158. 10.1111/j.1399-6576.2008.01847.x 19175575 10.1111/j.1399-6576.2008.01847.x

[CR10] Guérin L, Teboul JL, Persichini R, Dres M, Richard C, Monnet X (2015) Effects of passive leg raising and volume expansion on mean systemic pressure and venous return in shock in humans. Crit Care 19:411. 10.1186/s13054-015-1115-2 26597901 10.1186/s13054-015-1115-2 PMC4657233

[CR11] Hagiwara-Nagasawa M, Kambayashi R, Goto A, Chiba K, Wada T, Nunoi Y, Izumi-Nakaseko H, Takei Y, Matsumoto A, Lurie KG, Sugiyama A (2021) Effects of mechanical ventilation with expiratory negative airway pressure on porcine pulmonary and systemic circulation: mechano-physiology and potential application. J Physiol Sci 71:17. 10.1186/s12576-021-00801-5 34078262 10.1186/s12576-021-00801-5 PMC10717094

[CR12] He HW, Liu DW (2016) Passive leg raising in intensive care medicine. Chin Med J (Engl) 129:1755–1758. 10.4103/0366-6999.185866 27411467 10.4103/0366-6999.185866 PMC4960969

[CR13] Hinz J, Hahn G, Neumann P, Sydow M, Mohrenweiser P, Hellige G, Burchardi H (2003) End-expiratory lung impedance change enables bedside monitoring of end-expiratory lung volume change. Intensive Care Med 29:37–43. 10.1007/s00134-002-1555-4 12528020 10.1007/s00134-002-1555-4

[CR14] James R, Henning D, Smith J (2015) The use of impedance threshold devices in spontaneously breathing, hypotensive trauma patients. Trauma 17:102–108. 10.1177/1460408614539146

[CR15] Lima JNG, Fontes MS, Szmuszkowicz T, Isola AM, Maciel AT (2019) Electrical impedance tomography monitoring during spontaneous breathing trial: physiological description and potential clinical utility. Acta Anaesthesiol Scand 63:1019–1027. 10.1111/aas.13383 31066031 10.1111/aas.13383

[CR16] Longhini F, Maugeri J, Andreoni C, Ronco C, Bruni A, Garofalo E, Pelaia C, Cavicchi C, Pintaudi S, Navalesi P (2019) Electrical impedance tomography during spontaneous breathing trials and after extubation in critically ill patients at high risk for extubation failure: a multicenter observational study. Ann Intensive Care 9:88. 10.1186/s13613-019-0565-0 31410738 10.1186/s13613-019-0565-0 PMC6692788

[CR17] Lucato JJ, Tucci MR, Schettino GP, Adams AB, Fu C, Forti G Jr, de Carvalho CR, de Souza R (2005) Evaluation of resistance in 8 different heat-and-moisture exchangers: effects of saturation and flow rate/profile. Respir Care 50:636–643 15871758

[CR18] Ma J, Liu T, Wang Q, Xia X, Guo Z, Feng Q, Zhou Y, Yuan H (2023) Negative pressure pulmonary edema (review). Exp Ther Med 26:455. 10.3892/etm.2023.12154 37614417 10.3892/etm.2023.12154 PMC10443067

[CR19] Mauri T, Yoshida T, Bellani G, Goligher EC, Carteaux G, Rittayamai N, Mojoli F, Chiumello D, Piquilloud L, Grasso S, Jubran A, Laghi F, Magder S, Pesenti A, Loring S, Gattinoni L, Talmor D, Blanch L, Amato M, Chen L, Brochard L, Mancebo J (2016) Esophageal and transpulmonary pressure in the clinical setting: meaning, usefulness and perspectives. Intensive Care Med 42:1360–1373. 10.1007/s00134-016-4400-x 27334266 10.1007/s00134-016-4400-x

[CR20] McGee WT, Horswell JL, Calderon J, Janvier G, Van Severen T, Van den Berghe G, Kozikowski L (2007) Validation of a continuous, arterial pressure-based cardiac output measurement: a multicenter, prospective clinical trial. Crit Care 11:R105 17880692 10.1186/cc6125 PMC2556749

[CR21] Nicolò A, Sacchetti M (2023) Differential control of respiratory frequency and tidal volume during exercise. Eur J Appl Physiol 123:215–242 36326866 10.1007/s00421-022-05077-0

[CR22] Pinsky MR (2018) Cardiopulmonary interactions: physiologic basis and clinical applications. Ann Am Thorac Soc 15:S45–S48. 10.1513/AnnalsATS.201704-339FR 28820609 10.1513/AnnalsATS.201704-339FR PMC5822394

[CR23] Rocha NN, Silva PL, Battaglini D, Rocco PRM (2024) Heart-lung crosstalk in acute respiratory distress syndrome. Front Physiol 15:1478514 39493867 10.3389/fphys.2024.1478514 PMC11527665

[CR24] Schreiber T, Hüter L, Schwarzkopf K, Schubert H, Preussler N, Bloos F, Gaser E, Karzai W (2001) Lung perfusion affects preload assessment and lung water calculation with the transpulmonary double indicator method. Intensive Care Med 27:1814–1818 11810127 10.1007/s00134-001-1122-4

[CR25] Sigurdsson G, Yannopoulos D, McKnite SH, Sondeen JL, Benditt DG, Lurie KG (2006) Effects of an inspiratory impedance threshold device on blood pressure and short term survival in spontaneously breathing hypovolemic pigs. Resuscitation 68:399–404. 10.1016/j.resuscitation.2005.07.015 16455176 10.1016/j.resuscitation.2005.07.015

[CR26] Skytioti M, Søvik S, Elstad M (2018) Respiratory pump maintains cardiac stroke volume during hypovolemia in young, healthy volunteers. J Appl Physiol 124:1319–1325. 10.1152/japplphysiol.01009.2017 29494288 10.1152/japplphysiol.01009.2017

[CR27] Sobota V, Müller M, Roubík K (2019) Intravenous administration of normal saline may be misinterpreted as a change of end-expiratory lung volume when using electrical impedance tomography. Sci Rep 9:5775. 10.1038/s41598-019-42241-7 30962469 10.1038/s41598-019-42241-7 PMC6453964

[CR28] Spooner AJ, Corley A, Sharpe NA, Barnett AG, Caruana LR, Hammond NE, Fraser JF (2014) Head-of-bed elevation improves end-expiratory lung volumes in mechanically ventilated subjects: a prospective observational study. Respir Care 59:1583–1589. 10.4187/respcare.02733 24847096 10.4187/respcare.02733

[CR29] Takahashi K, Toyama H, Funahashi Y, Kawana S, Ejima Y, Kikuchi K, Ishikawa T, Yamauchi M (2022) Influence of respiratory gas density on tidal volume during mechanical ventilation: a laboratory investigation and observational study in children. Tohoku J Exp Med 256:271–281. 10.1620/tjem.2022.j003 35296568 10.1620/tjem.2022.J003

[CR30] Takahashi K, Toyama H, Ejima Y, Yang J, Kikuchi K, Ishikawa T, Yamauchi M (2023a) Endotracheal tube, by the venturi effect, reduces the efficacy of increasing inlet pressure in improving pendelluft. PLoS ONE 18:e0291319. 10.1371/journal.pone.0291319 37708106 10.1371/journal.pone.0291319 PMC10501657

[CR31] Takahashi K, Toyama H, Kubo R, Yoshida N, Ejima Y, Kikuchi K, Ishikawa T, Yamauchi M (2023b) Effectiveness of substantial shortening of the endotracheal tube for decreasing airway resistance and increasing tidal volume during pressure-controlled ventilation in pediatric patients: a prospective observational study. J Clin Monit Comput 37:1513–1519. 10.1007/s10877-023-01038-w 37289350 10.1007/s10877-023-01038-w

[CR32] van den Bosch OFC, Alvarez-Jimenez R, de Grooth HJ, Girbes ARJ, Loer SA (2021) Breathing variability-implications for anaesthesiology and intensive care. Crit Care 25:280 34353348 10.1186/s13054-021-03716-0 PMC8339683

[CR33] Wisse JJ, Goos TG, Jonkman AH, Somhorst P, Reiss IKM, Endeman H, Gommers D (2024) Electrical impedance tomography as a monitoring tool during weaning from mechanical ventilation: an observational study during the spontaneous breathing trial. Respir Res 25:179. 10.1186/s12931-024-02801-6 38664685 10.1186/s12931-024-02801-6 PMC11044327

[CR34] Yoshida T, Fujino Y, Amato MBP, Kavanagh BP (2017) Fifty years of research in ARDS. Spontaneous breathing during mechanical ventilation. Risks, mechanisms, and management. Am J Respir Crit Care Med 195:985–992. 10.1164/rccm.201604-0748CP 27786562 10.1164/rccm.201604-0748CP

[CR35] Zhang Y, Wang Y, Shi J, Hua Z, Xu J (2019) Cardiac output measurements via echocardiography versus thermodilution: a systematic review and meta-analysis. PLoS One 14:e0222105 31581196 10.1371/journal.pone.0222105 PMC6776392

[CR36] Zhao Z, Peng SY, Chang MY, Hsu YL, Frerichs I, Chang HT, Möller K (2017) Spontaneous breathing trials after prolonged mechanical ventilation monitored by electrical impedance tomography: an observational study. Acta Anaesthesiol Scand 61:1166–1175. 10.1111/aas.12959 28832898 10.1111/aas.12959

