# Supplementary material for: Inspiratory effort increases blood volume in the thoracic cavity and decreases end-expiratory lung impedance: a preliminary prospective study
Source: Eur J Appl Physiol. 2025 Apr 3;125(9):2479–86. doi: 10.1007/s00421-025-05767-5 (PMC12423182; doi:10.1007/s00421-025-05767-5)
Supplement: Supplementary file 1 — Supplementary file1 (PDF 203 KB) [file 421_2025_5767_MOESM1_ESM.pdf]

Respiratory parameters of each participant at each step.

Inspiratory effort increases blood volume in the thoracic cavity and decreases end-expiratory lung impedance: a preliminary prospective study

Kazuhiro Takahashi[1], Ayaka Koyama[1], Daisuke Irimada[1], Akihiro Kanaya[1], Daisuke Konno[1], Yu Kaiho[1], Yusuke Takei[1], Kazutomo Saito[1], Yutaka Ejima[1], Masanori Yamauchi[1]

[1] Anesthesiology and Perioperative Medicine, Tohoku University Graduate School of Medicine, Sendai, Japan

Corresponding author: Kazuhiro Takahashi, kazuhiro.takahashi.c4@tohoku.ac.jp

Online Resource 1. Respiratory parameters of each participant at each step**.**

The mean end-expiratory lung impedance (EELI), mean airway pressure and maximal inspiratory flow rate of each participant at each step are shown in the table below. The average EELI value for 24 breaths in the control steps was used as the reference for each participant, and the difference between the measured value and each participant's reference value was used for statistical analysis (ΔEELI) in the main text.

No.		C	PLR	C 2	3 mm	C 3	2 mm	C 4	reference value
1	EELI	-28.7	-40.6	-33.6	-38.2	-35.2	-38.9	-33.5	-32.7
	Mean Pres.	-0.1	-0.1	0.0	-5.3	-0.1	-7.5	0.0	
	Max. Flow	39.5	42.3	41.8	24.9	46.9	16.1	44.3	
2	EELI	-78.5	-90.8	-90.6	-108.2	-93.2	-110.3	-97.4	-89.9
	Mean Pres.	-0.2	0.0	-0.1	-4.3	-0.1	-4.9	-0.1	
	Max. Flow	39.4	39.7	37.3	19.9	39.6	11.0	40.3	
3	EELI	-58.8	-71.9	-54.2	-62.1	-46.5	-57.4	-46.2	-51.4
	Mean Pres.	0.1	0.0	0.0	-1.9	-0.1	-3.8	-0.1	
	Max. Flow	32.6	43.2	36.5	13.6	40.1	10.8	36.1	
4	EELI	-17.9	-31.5	-19.8	-24.2	-16.9	-30.3	-11.2	-16.4
	Mean Pres.	0.0	0.0	-0.1	-1.6	-0.3	-4.3	-0.1	
	Max. Flow	23.8	23.5	23.0	13.5	37.0	10.7	38.6	
5	EELI	-57.2	-75.3	-51.0	-61.3	-54.1	-58.3	-54.2	-54.1
	Mean Pres.	0.0	0.1	-0.1	-1.6	0.0	-2.7	-0.4	
	Max. Flow	34.7	38.2	38.3	15.4	42.1	10.3	42.7	
6	EELI	-131.4	-136.9	-135.0	-149.6	-135.8	-141.9	-128.0	-132.6
	Mean Pres.	-0.3	-0.2	-0.3	-6.3	0.0	-8.4	-0.2	
	Max. Flow	71.3	67.9	70.7	29.4	65.7	16.8	68.9	
7	EELI	-54.4	-66.9	-63.2	-81.1	-65.3	-92.4	-71.6	-63.6
	Mean Pres.	0.0	-0.3	-0.1	-6.9	-0.2	-5.6	-0.2	
	Max. Flow	51.3	59.7	53.9	25.7	63.1	12.8	44.6	
8	EELI	-67.8	-81.0	-90.2	-104.5	-84.9	-94.0	-72.5	-78.8
	Mean Pres.	0.0	0.1	0.1	-5.3	-0.1	-9.8	0.0	
	Max. Flow	30.6	29.4	26.7	23.7	43.0	20.4	39.9	
9	EELI	-39.8	-64.5	-58.5	-77.2	-58.9	-82.1	-62.5	-54.9
	Mean Pres.	0.1	0.0	0.1	-4.1	-0.2	-7.8	-0.5	
	Max. Flow	31.2	40.0	41.6	21.2	69.4	16.0	84.7	
10	EELI	-6.7	-30.8	3.0	-7.4	2.6	-3.5	4.7	0.9
	Mean Pres.	-0.1	0.2	0.0	-2.5	-0.1	-4.4	-0.1	
	Max. Flow	57.8	54.4	51.1	26.6	50.7	22.5	54.3	
11	EELI	-45.2	-40.6	-57.0	-70.3	-52.5	-84.1	-57.1	-53.0
	Mean Pres.	-0.2	-0.1	-0.2	-7.0	-0.4	-10.4	-0.3	
	Max. Flow	50.5	50.5	49.5	30.6	59.6	21.0	60.1	

C, control; Mean Pres, mean airway pressure; Max. Flow, maximal inspiratory flow rate.
