# Supplementary material for: Inspiratory effort increases blood volume in the thoracic cavity and decreases end-expiratory lung impedance: a preliminary prospective study
Source: Eur J Appl Physiol. 2025 Apr 3;125(9):2479–86. doi: 10.1007/s00421-025-05767-5 (PMC12423182; doi:10.1007/s00421-025-05767-5)
Supplement: Supplementary file 2 — Supplementary file2 (PDF 186 KB) [file 421_2025_5767_MOESM2_ESM.pdf]

Mean ΔEELI during the measurements

Inspiratory effort increases blood volume in the thoracic cavity and decreases end-expiratory lung impedance: a preliminary prospective study

Kazuhiro Takahashi[1], Ayaka Koyama[1], Daisuke Irimada[1], Akihiro Kanaya[1], Daisuke Konno[1], Yu Kaiho[1], Yusuke Takei[1], Kazutomo Saito[1], Yutaka Ejima[1], Masanori Yamauchi[1]

[1] Anesthesiology and Perioperative Medicine, Tohoku University Graduate School of Medicine, Sendai, Japan

Corresponding author: Kazuhiro Takahashi, kazuhiro.takahashi.c4@tohoku.ac.jp

Online Resource 2. Mean ΔEELI during the measurements.

From left to right, the seven box whiskers indicate the first control step (white monochrome), PLR (gray monochrome), second control step (narrow vertical stripes on white background), 3 mm (black monochrome), third control step (thick vertical stripes on white background), 2 mm (narrow vertical stripes on gray background), and fourth control step (diagonal stripes on white background). For each box whisker, the box represents the interquartile range (IQR) containing the middle 50% of the data. The line inside the box indicates the median, the crossed mark indicates the average value, and the whiskers show the rest of the distribution, excluding outliers (points beyond whiskers). Black circles indicate data from individual participants.

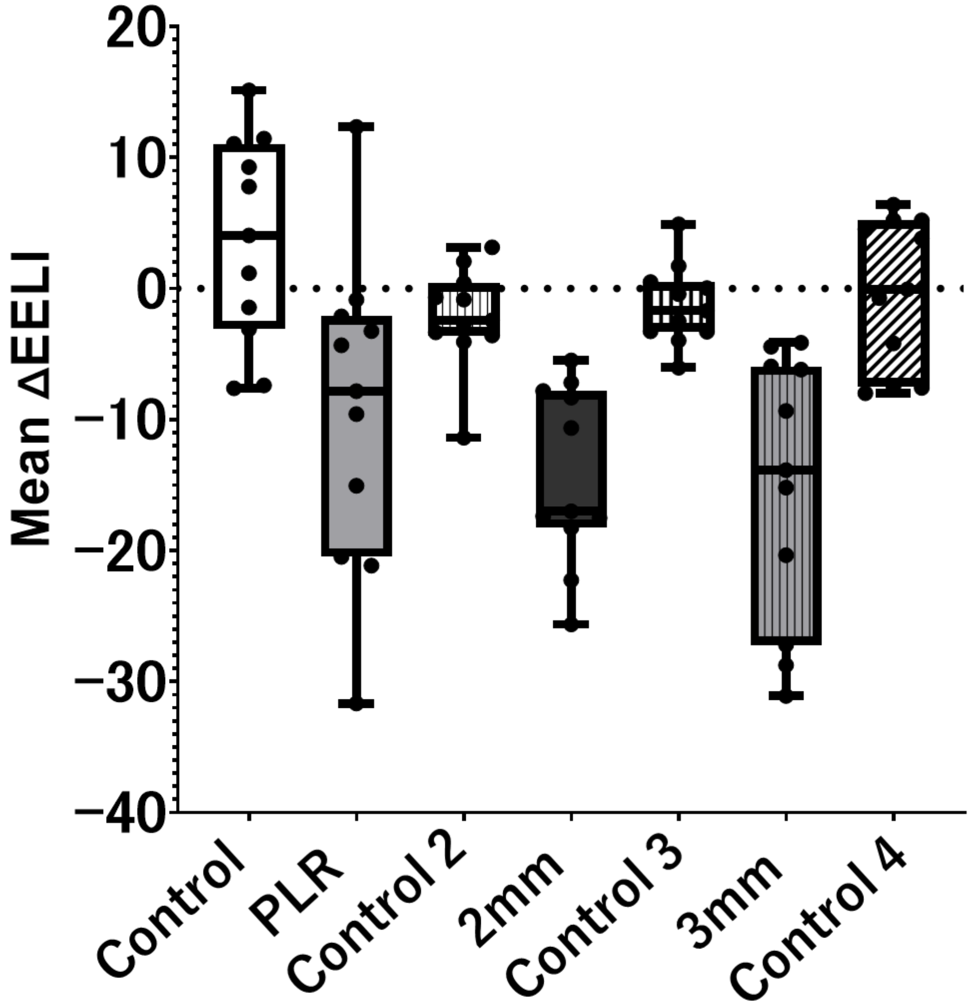

20
10
0
−10
−20
−30
−40
Mean ΔEELI
Control
PLR
Control 2
2mm
Control 3
3mm
Control 4
